# Predicting Durable Responses to Immune Checkpoint Inhibitors in Non-Small-Cell Lung Cancer Using a Multi-Feature Model

**DOI:** 10.3389/fimmu.2022.829634

**Published:** 2022-04-22

**Authors:** Lei Wang, Hongbing Zhang, Chaohu Pan, Jian Yi, Xiaoli Cui, Na Li, Jiaqian Wang, Zhibo Gao, Dongfang Wu, Jun Chen, Jizong Jiang, Qian Chu

**Affiliations:** ^1^ Department of Oncology, Tongji Hospital, Tongji Medical College, Huazhong University of Science and Technology, Wuhan, China; ^2^ Department of Lung Cancer Surgery, Tianjin Lung Cancer Institute, Tianjin Medical University General Hospital, Tianjin, China; ^3^ Department of Medicine, YuceBio Technology Co., Ltd, Shenzhen, China; ^4^ The First Affiliated Hospital, Jinan University, Guangzhou, China; ^5^ Zhuhai Institute of Translational Medicine, Zhuhai People’s Hospital (Zhuhai Hospital Affiliated with Jinan University), Jinan University, Zhuhai, China; ^6^ The Biomedical Translational Research Institute, Faculty of Medical Science, Jinan University, Guangzhou, China

**Keywords:** immune checkpoint inhibitors, durable responses, multi-feature model, genetic biomarkers, non-small cell lung cancer, cancer immunity and immunotherapy

## Abstract

Due to the complex mechanisms affecting anti-tumor immune response, a single biomarker is insufficient to identify patients who will benefit from immune checkpoint inhibitors (ICIs) treatment. Therefore, a comprehensive predictive model is urgently required to predict the response to ICIs. A total of 162 non-small-cell lung cancer (NSCLC) patients undergoing ICIs treatment from three independent cohorts were enrolled and used as training and test cohorts (training cohort = 69, test cohort1 = 72, test cohort2 = 21). Eight genomic markers were extracted or calculated for each patient. Ten machine learning classifiers, such as the gaussian process classifier, random forest, and support vector machine (SVM), were evaluated. Three genomic biomarkers, namely tumor mutation burden, intratumoral heterogeneity, and loss of heterozygosity in human leukocyte antigen were screened out, and the SVM_poly method was adopted to construct a durable clinical benefit (DCB) prediction model. Compared with a single biomarker, the DCB multi-feature model exhibits better predictive value with the area under the curve values equal to 0.77 and 0.78 for test cohort1 and cohort2, respectively. The patients predicted to have DCB showed improved median progression-free survival (mPFS) and median overall survival (mOS) than those predicted to have non-durable clinical benefit.

## 1 Introduction

Lung cancer is one of the most commonly diagnosed malignant tumors, the majority of which are non-small-cell lung cancers (NSCLCs) (1). Among all cancer types, the incidence and mortality of lung cancer are ranked second and first, respectively (2). Immune checkpoint inhibitors (ICIs), including anti-cytotoxic T lymphocyte-associated antigen-4 (CTLA4) and anti-programmed cell death protein 1 (PD-1)/programmed death-ligand 1 (PD-L1), have dramatically altered the treatment landscape of NSCLC (3–8). However, only a small subset of patients benefit from ICIs treatment, and some may even suffer from immune-related adverse events, requiring treatment discontinuation (9–12). Therefore, identification of more effective predictive biomarkers that can guide the treatment decision making with ICIs is significantly important and urgent.

Multiple clinical trials have verified that both PD-L1 expression level and tumor mutation burden (TMB) can predict the efficacy of ICIs in NSCLC (13–18). Recently, several novel genome-related biomarkers such as intratumoral heterogeneity (ITH), tumor neoantigen burden (TNB), loss of heterozygosity in human leukocyte antigen (HLA LOH), and HLA-I evolutionary divergence (HED), have been demonstrated to be associated with response to ICIs in patients (19–23). These single indicators can distinguish responders from non-responders to a certain extent, although their sensitivity and accuracy need to be further improved. For instance, some NSCLC patients with high TMB cannot benefit from immunotherapy (24).

Our objective was to construct a robust predictive model to predict durable response to ICIs in NSCLC patients based on multiple genomic features, and to assess its potential for clinical decision-making guidance in cancer treatment with ICIs.

## 2 Materials and Methods

### 2.1 Study Cohorts

Genomic and clinical data of 69 and 72 NSCLC patients undergoing ICIs treatments were obtained from published cohorts, of which 69 NSCLC patients were used as the training cohort and 72 NSCLC patients were used as the test cohort1 (19, 25).

To further evaluate the accuracy and effectiveness of the multi-feature model, 21 patients treated with ICIs, collected from January 2018 to May 2020, were enrolled in this study and used as test cohort2.

Durable clinical benefit (DCB) was defined as complete response (CR), partial response (PR), or stable disease (SD) that lasted for ≥ 24 weeks, and non-durable benefit (NDB) was defined as progressive disease (PD) or SD that lasted for < 24 weeks.

Among the 162 NSCLC patients included in this study, 69% (111/162) were lung adenocarcinoma. The number of patients with DCB and NDB was 61 and 101, respectively. Detailed clinical information of patients is summarized in **Supplementary Tables 1**–**3**.

In addition, genomic and clinical data of 120 melanoma patients undergoing ICIs treatments were obtained from published cohort to further evaluate the accuracy and effectiveness of the multi-feature model (26). The patients with CR or PR were 55, and the patients with PD were 65. DCB was defined as CR and PR, and NDB was defined as PD. Detailed clinical information of patients is summarized in **Supplementary Table 4**.

### 2.2 Next-Generation Sequencing (NGS) and Mutation Analysis

Genomic profiling was performed on tumor tissues and matched peripheral blood samples. First, we used the GeneReadDNA FFPE kit (Qiagen) and Qiagen DNA blood mini kit (Qiagen) to extract DNA from tumor specimens and blood, respectively. Then, the extracted DNA was amplified, purified, and analyzed using an NGS panel (YuceOne™ Plus, Yucebio, China).

Sequencing reads with > 10% N rate and/or > 10% bases with a quality score < 20 were filtered using SOAPnuke (Version 1.5.6) (27). Somatic single nucleotide variants and insertions and deletions (indels) were detected using VarScan (Version 2.4) (28). Next, an in-house method was applied to filter possible false-positive mutations. Finally, SnpEff (Version 4.3) was used to functionally annotate the mutations detected in the tumor samples (29).

### 2.3 Evaluation of Genomic Biomarkers

#### 2.3.1 Evaluation of Tumor Mutation Burden

TMB was determined as the number of all nonsynonymous mutations and indels per megabase of the genome examined, and the cut-off value for TMB-high and TMB-low was defined as the median TMB.

#### 2.3.2 Evaluation of HLA Typing, HED, and Somatic HLA Loss

HLA typing of the paired peripheral blood and tumor samples was performed from whole-exome sequencing data using POLYSOLVER (v1.0) (30) and OptyType (v1.3.2) (31). A scoring algorithm was then used to integrate the results that were used for further analysis (32). HED was calculated as previously described (33). The mean HED of patients was calculated as the mean of divergences at HLA_A, HLA_B, and HLA_C, and the bioinformatic tool LOHHLA with the default program settings was used to determine their maintenance or loss in the tumor (21).

#### 2.3.3 Evaluation of TNB

All nonsynonymous mutations and indels were translated into 21-mer peptide sequences using in-house software centered on the mutated amino acid. Then, the 21-mer peptide was used to create a 9- to 11-mer peptide *via* a sliding window approach for the prediction of MHC class I binding affinity. Next, NetMHCpan (v3.0) was used to predict the binding strength of the mutated peptides to patient-specific HLA alleles (34). A peptide with predicted binding affinity to any HLA allele with an IC50 < 500 nM was selected. If several selected peptides were generated from the same mutation, they were counted as one neoantigen. TNB was determined as the number of putative neoantigens per megabase of the genome.

#### 2.3.4 Calculation of Copy Number Variants (CNV) and ITH

CNVs were called using CNVkit (v0.8.1) to compare the exome-wide profile between tumors and matched peripheral blood (35). Allele-specific copy number and tumor purity were assessed using the ascatNgs (v3.1.0). PyClone (v0.13.0) was used to infer the cancer cell fraction (CCF) of mutations in tumors. ITH was calculated using a previously developed method (19).

### 2.4 Construction of the Multi-Feature Model

Ten classifiers, including K-Nearest Neighbors (KNN), Logistic Regression (LR), Random Forest (RF), Gradient Boosting Classifier (GBC), Decision Tree Classifier (DTC), Extra Tree Classifier (ETC), Gaussian Process Classifier (GPC), support vector machine (SVM)_poly, SVM_rbf, and SVM_liner, were evaluated to construct a multi-feature model with three genomic biomarkers to predict the efficacy of ICIs in the training cohort. The GridSearchCV and cross_val_score packages in Sklearn (version 0.24.1.) were used to iteratively optimize the RF, GBC, and DTC algorithms. The default parameters were adopted for the other algorithms. The predicted efficacy of these algorithms was calculated using the area under the receiver operating characteristic (ROC) curve. In addition, we evaluated the accuracy, specificity, sensitivity, positive predictive value, and negative predictive value of these algorithms in the training cohort.

### 2.5 Statistical Methods

An ROC curve was generated to evaluate diagnostic accuracy and the area under the curve was calculated to measure the discriminatory ability of potential biomarkers. Progression-free survival (PFS) was analyzed using the Kaplan–Meier method and log-rank test.

## 3 Results

### 3.1 Single Biomarker Has Limited Ability to Distinguish the Responders From the Non-Responders

**Figure 1:**
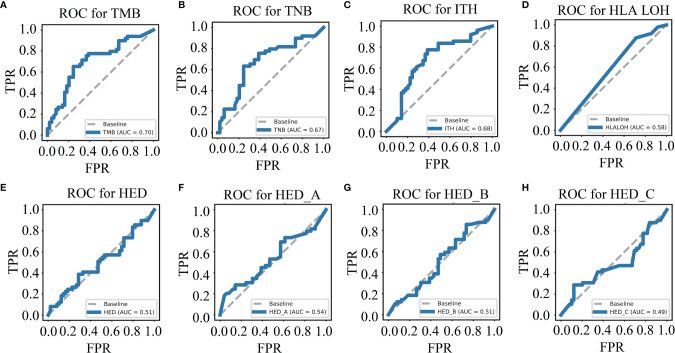
Single biomarker has limited ability to distinguish the responders from the non-responders. ROC curves for TMB **(A)**, TNB **(B)**, ITH **(C)**, HLA LOH **(D)**, HED **(E)**, HED_A **(F)**, HED_B **(G)**, and HED_C **(H)** in the training cohort. TMB, tumor mutation burden; ITH, intratumoral heterogeneity; HLA LOH, loss of heterozygosity in human leukocyte antigen; HFD, HLA-I evolutionary divergence; ROC, receiver operating characteristic.

To evaluate the predictive ability of a single biomarker for ICIs response, eight genomic biomarkers (TMB, TNB, ITH, HLA LOH, HED, HED_A, HED_B and HED_C) were analyzed, respectively. First, we winsorized and normalized the data in the training cohort (**Supplementary Figure 1**). The receiver operating characteristic (ROC) curves of the individual biomarkers were plotted and their predictive ability was measured using the area under the curve (AUC) value. As shown in **Figures 1A–H**, the accuracy and effectiveness of TMB, TNB, and ITH (AUC = 0.70, 0.67, and 0.68, respectively) were higher than those of HLA LOH, HED, HED_A, HED_B, and HED_C (AUC = 0.58, 0.51, 0.54, 0.51, and 0.49, respectively); however, none of these values exceeded 0.7, suggesting that a single biomarker was not effective enough to precisely distinguish responders from non-responders treated with ICIs.

### 3.2 Feature Selection and Model Evaluation

**Figure 2:**
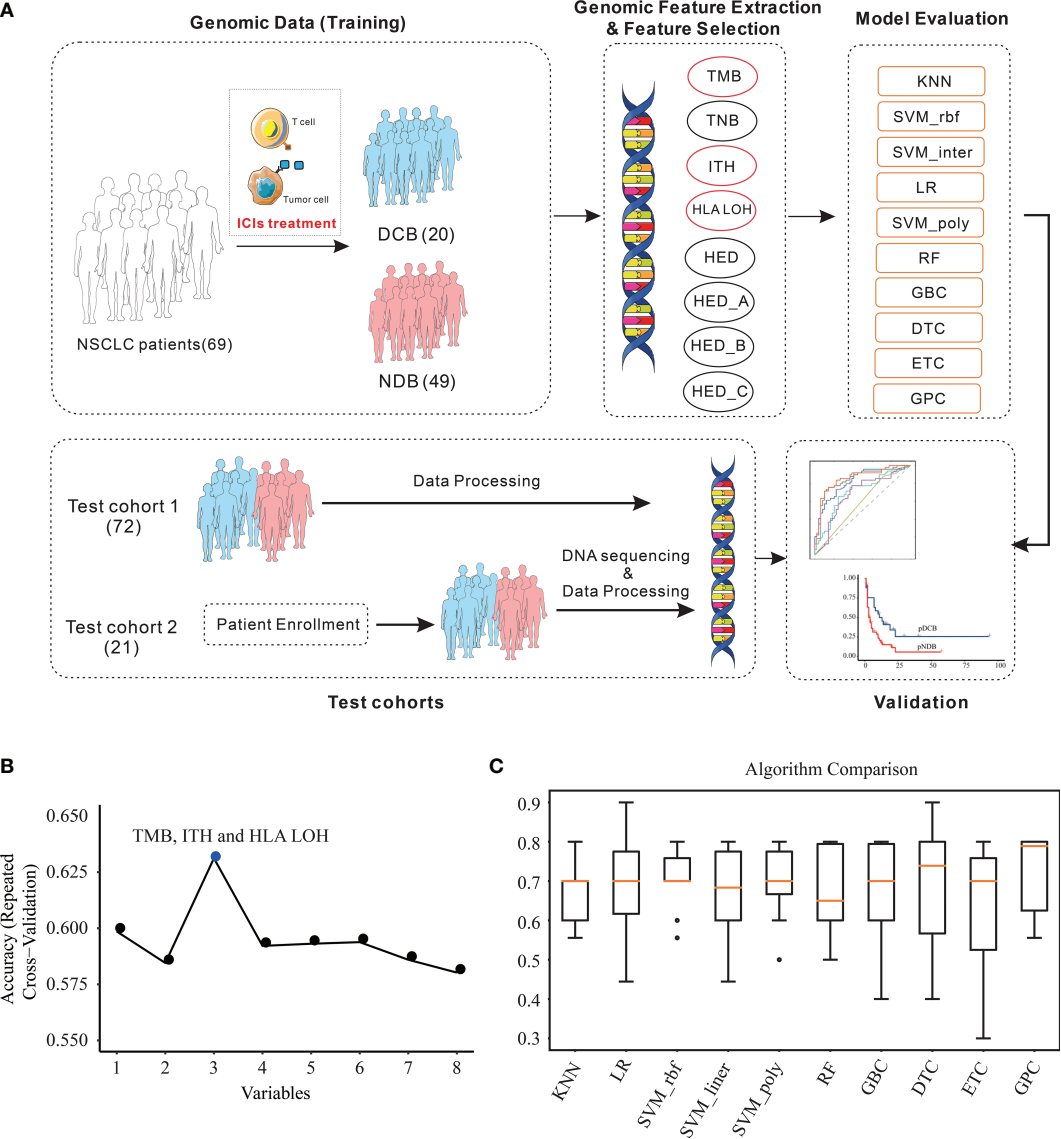
Feature combination selection and performance evaluation. **(A)** Workflow of the study. **(B)** Feature combination selection with 10-fold cross-validation. **(C)** Comparison of the efficacy of different algorithms in the training cohort with the selected features. KNN, K-nearest neighbors; LR, logistic regression; RF, random forest; GBC, gradient boosting classifier; DTC, decision tree classifier; ETC, extra tree classifier; GPC, Gaussian process classifier; SVM, support vector machine.

Due to the limited predictive ability of a single biomarker, the predictive ability of the multi-feature model was further investigated and the detailed processes were shown in **Figure 2A**.

To avoid model overfitting, cross-validated recursive feature elimination was applied to select features from eight biomarkers (TMB, TNB, ITH, HLA LOH, HED, HED_A, HED_B, and HED_C). As shown in **Figure 2B** and **Table 1**, three of them, TMB, ITH, and HLA LOH, were screened to predict the efficacy of ICIs in the training cohort.

**Table 1:** List of the optimal combination from eight features.

Biomarker	Feature Selection	Ranking
TMB	True	1
ITH	True	1
HLA LOH	True	1
HED_C	False	2
TNB	False	3
HED_B	False	4
HED_A	False	5
HED	False	6

Based on the three selected features, the efficacy of different algorithms (KNN, LR, SVM_rbf, SVM_ linear, SVM_poly, RF, GBC, DTC, ETC, and GPC) were evaluated and compared. The best-performing hyperparameters were determined by 10-fold cross-validation in the training cohort. As shown in **Figure 2C** and **Table 2**, the top four algorithms for accuracy were GPC, DTC, SVM_rbf, and SVM_poly, while the top four algorithms for variance were KNN, SVM_rbf, SVM_poly, and GPC. After comprehensive consideration of the accuracy and variance, the GPC, SVM_rbf, and SVM_poly algorithms were selected for further analysis.

**Table 2:** List of the accuracy and variance of different algorithms with 10-fold cross-validation in the training cohort.

Algorithm	Accuracy	Variance
KNN	0.66	0.08
LR	0.68	0.13
SVM_rbf	0.70	0.08
SVM_linear	0.66	0.12
SVM_poly	0.69	0.09
RF	0.67	0.11
GBC	0.67	0.13
DTC	0.70	0.14
ETC	0.65	0.16
GPC	0.72	0.10

### 3.3 Algorithm Selection for Multi-Feature Model Construction

**Figure 3:**
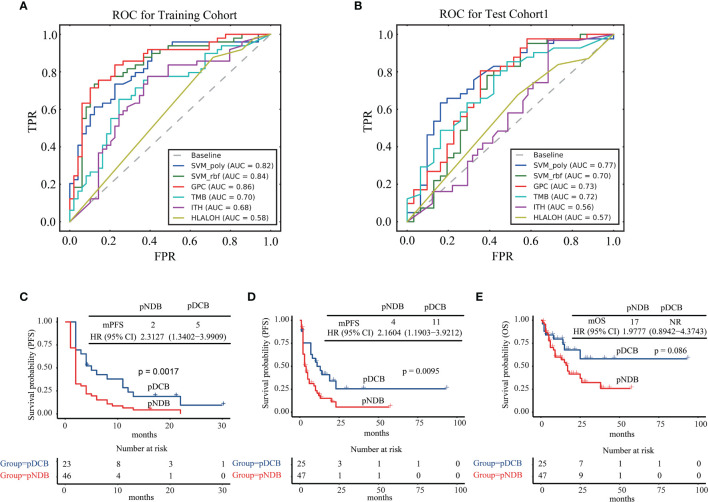
Algorithm selection for multi-feature model construction. **(A)** ROC curves for SVM_poly, SVM_rbf, GPC, TMB, ITH, and HLA LOH in the training cohort. **(B)** ROC curves for SVM_poly, SVM_rbf, GPC, TMB, ITH, and HLA LOH in test cohort1. **(C)** Kaplan–Meier curves of PFS comparing pDCB with pNDB in the training cohort. **(D)** Kaplan–Meier curves of PFS comparing pDCB with pNDB in test cohort1. **(E)** Kaplan–Meier curves of OS comparing pDCB with pNDB in test cohort1. pDCB, patients predicted to have durable clinical benefit; pNDB, patients predicted to have no durable benefit; PFS, progression-free survival; OS, overall survival.

The algorithms of GPC, SVM_rbf, and SVM_poly were next assessed by their ability to predict the efficacy of patients with ICIs in the training cohort and test cohort1. As shown in **Figures 3A, B**, the AUC values of GPC, SVM_rbf, and SVM_poly algorithms were higher than those predicted by a single biomarker. Moreover, compared with the GPC and SVM_rbf algorithms, the SVM_poly algorithm was stabler in the training cohort and test cohort1, and thus was more suitable to be selected to construct the multi-feature model.

Next, the median progression-free survival (mPFS) and median overall survival (mOS) in the training cohort and test cohort1 were analyzed with the multi-feature model. The results showed that patients who were predicted to have durable clinical benefit (pDCB) had longer mPFS and mOS than those predicted to have no durable benefit (pNDB) (**Figures 3C–E**).

### 3.4 Validation of the Multi-Feature Model in Patients Enrolled in Test Cohort2

**Figure 4:**
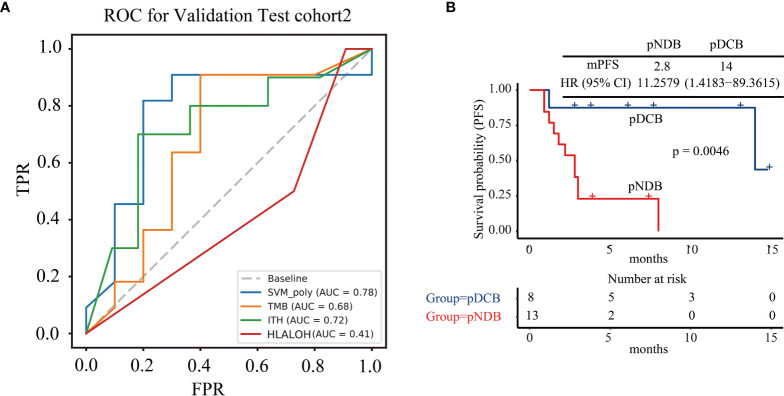
The multi-feature model could effectively predict response to ICIs treatment in test cohort2. **(A)** ROC curves for multi-feature model, TMB, ITH, and HLA LOH in test cohort2. **(B)** Kaplan–Meier curves of PFS comparing pDCB with pNDB in test cohort2.

Twenty-one NSCLC patients treated with ICIs enrolled in this study were used for further validation of the multi-feature model. Consistent with the above results, the AUC value predicted by the multi-feature model was higher than those predicted by single biomarker (**Figure 4A**). Furthermore, the mPFS of the pDCB subgroup was significantly longer than that of the pNDB subgroup (**Figure 4B**). In conclusion, the multi-feature model is able to distinguish pDCB from pNDB.

## 4 Discussion

ICIs have achieved great success in the treatment of NSCLC, and several biomarkers have been developed to help clinicians make treatment choices; however, the predictive accuracy of these biomarkers is finite (24, 36). In this study, a comprehensive analysis of NSCLC samples was carried out to investigate the role of a multi-feature model composed of TMB, ITH, and HLA LOH in determining the response to ICIs. Our study showed that the accuracy of this multi-feature model was higher than that of any single biomarker, and the mPFS and mOS of pDCB patients were longer than those of pNDB patients predicted by the multi-feature model. Finally, whole-exome sequencing data from 21 NSCLC patients treated with ICIs were applied to further validate the model, and the same results were obtained.

Several studies have integrated multiple indicators to predict the efficacy of ICIs in NSCLC patients. Shi et al. integrated genomic profiling, TMB, and the expression level of PD-L1 to predict the efficacy of ICIs and reported that *KMT2C/KRAS/TP53* co-mutation could serve as a biomarker to identify the best responders to ICIs therapy; however, the percentage of durable clinical benefit was only about 50% (37). Lin et al. also constructed a comprehensive predictive classifier model based on epidermal growth factor receptor and AT‐rich interaction domain 1B status, smoking history, treatment type, and PD-L1 score and found that patients with low-risk scores showed improved PFS compared to those with high-risk scores, while the AUC value of 6-month PFS was only 0.75 (38). In addition, these prediction models required multiple detection techniques, such as targeted panel sequencing and IHC of PD-L1, which need more tumor samples and costs. Therefore, in terms of the availability of clinical samples and the costs of treatment, we hoped that multiple features could be obtained through one detection technology. Targeted panel sequencing has been demonstrated in clinical application. As the costs of sequencing decreased, the clinical application of targeted panel sequencing gradually increased. More importantly, from targeted panel sequencing, multiple genomic features could be analyzed. Therefore, the multi-feature model based on genomic markers was developed. In our study, the AUC values of the multi-feature model in the training cohort, test cohort1 and test cohort2 were 0. 82, 0.77, and 0.78, respectively, which proved that our model is more effective in predicting the efficacy of NSCLC patients treated with ICIs. In order to further expand the application of this model, the predictive ability of this model in melanoma was also analyzed. As shown in **Figure S2**, the AUC value was 0.6, which indicated that the model may be unsuitable for melanoma.

In summary, we have constructed a multi-feature model that can effectively predict the efficacy of NSCLC patients treated with ICIs, which can help in clinical decision-making. In addition, patients with pDCB could be considered as more suitable candidates for treatment with ICIs. Ongoing intense work, especially prospective large cohorts, is needed to further validate and optimize our model.

## Data Availability Statement

The datasets presented in this study can be found in online repositories, *via* the following link: https://ngdc.cncb.ac.cn/gsa-human/browse/HRA001966.

## Ethics Statement

The studies involving human participants were reviewed and approved by YuceBio Ethics Committee for research in health. The patients/participants provided their written informed consent to participate in this study.

## Author Contributions

Conceptualization, DW, JC, JJ, QC and ZG. Methodology, LW, HZ, CP, JY and JW. Data analysis, LW, HZ, CP, JY and XC. Wrote the manuscript, LW, HZ, CP, JY and XC. Revised the manuscript, LW, HZ, CP and NL. All authors contributed to the article and approved the submitted version.

## Funding

This study was supported by Natural Science Foundation of China (NO. 62131009 and 82072597).

## Conflict of Interest

Author CP, JY, XC, NL, JW, ZG and DW were employed by the company YuceBio Technology Co., Ltd.

The remaining authors declare that the research was conducted in the absence of any commercial or financial relationships that could be construed as a potential conflict of interest.

## Publisher’s Note

All claims expressed in this article are solely those of the authors and do not necessarily represent those of their affiliated organizations, or those of the publisher, the editors and the reviewers. Any product that may be evaluated in this article, or claim that may be made by its manufacturer, is not guaranteed or endorsed by the publisher.

## Supplementary Material

Supplementary Figure 1 Data winsorization and normalization in the training cohort. **(A)** Data of HED, HED_A, HED_B, HED_C, TMB, ITH and TNB before winsorization. **(B)** Data of HED, HED_A, HED_B, HED_C, TMB, ITH and TNB after winsorization. **(C)** Data of HED, HED_A, HED_B, HED_C, TMB, ITH and TNB after normalization.

Supplementary Figure 2 ROC curves for multi-feature model, TMB, ITH, and HLA LOH in cohort of melanoma.
