# Supplementary figures and images for: Predicting Durable Responses to Immune Checkpoint Inhibitors in Non-Small-Cell Lung Cancer Using a Multi-Feature Model
Source: Front Immunol. 2022 Apr 22;13:829634. doi: 10.3389/fimmu.2022.829634 (PMC9072668; doi:10.3389/fimmu.2022.829634)

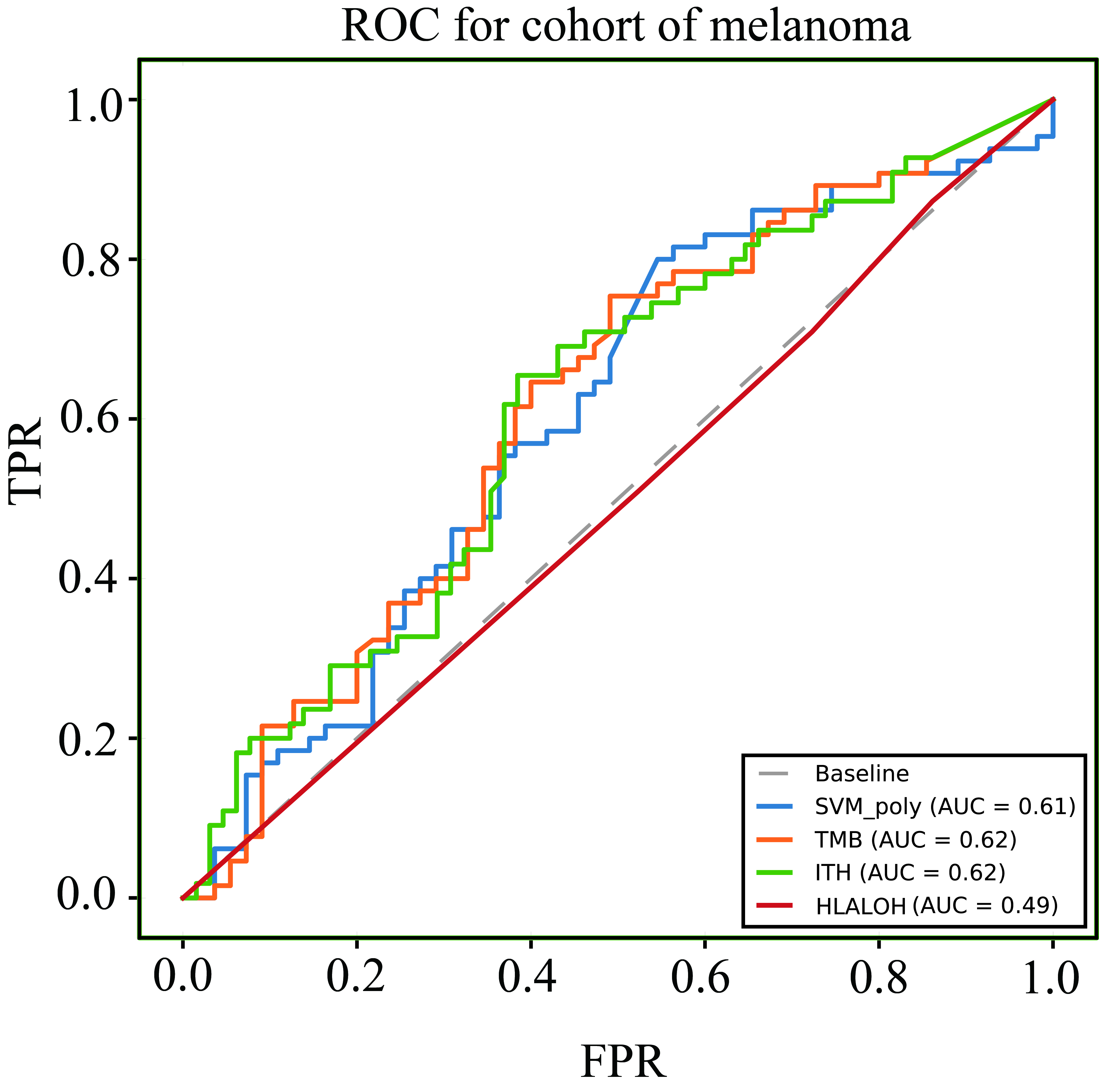

Supplement: Supplementary Figure 2 — ROC curves for multi-feature model, TMB, ITH, and HLA LOH in cohort of melanoma. [file Image_2.jpeg]
